# Effect of Steroids on the Progression of Alzheimer's Dementia: A Retrospective Chart Review

**DOI:** 10.1002/agm2.70004

**Published:** 2025-02-18

**Authors:** Julijana Zoran Conic, Alexandra Chetty, Lily Chen, Audrey Marsh, Sean Barry, Rodney Pattabhi, Thomas Reske, Erwin Aguilar, Lobna Ali

**Affiliations:** ^1^ Section of Geriatric Medicine, Department of Internal Medicine, LSU Health Sciences Center School of Medicine in New Orleans New Orleans Louisiana USA; ^2^ Section of General Internal Medicine, Department of Internal Medicine, LSU Health Sciences Center School of Medicine in New Orleans New Orleans Louisiana USA; ^3^ Section of Nephrology and Hypertension, Department of Internal Medicine, LSU Health Sciences Center School of Medicine in New Orleans New Orleans Louisiana USA; ^4^ Section of Geriatric Medicine and Hematology/Oncology, Department of Internal Medicine, LSU Health Sciences Center School of Medicine in New Orleans New Orleans Louisiana USA

## Abstract

**Objectives:**

Alzheimer's disease (AD) is a prevalent age‐related neurodegenerative disease that affects millions of individuals in the United States. Neuroinflammation is a driver of the neurodegenerative changes that characterize AD, prompting interest in how inflammation can be modulated for treatment and prevention.

**Methods:**

ICD‐10 codes were quarried from electronic medical records to identify patients diagnosed with AD from 2012 to 2020. The patients were then divided into those who used systemic steroids and those who did not before the progression of their disease. Data on medication prescribed was used to measure the disease's progression. Clinical findings and laboratory results were collected to build a propensity score. Patients were followed until disease progression, death, or the last available visit. Kaplan–Meier curves and hazard ratios adjusted for the propensity score were used to compare the two groups.

**Results:**

Of the 459 patients identified, 77 were included in the study, and 13 used steroids. Of the 77 patients included in the study, 59 had progression of their disease, and of those, five used steroids. The median time to progression was 408.00 (191.00, 979.00) days for the overall sample. The hazard ratio (HR) comparing the group using steroids to those not using steroids was 0.26 with a 95% CI of (0.1013, 0.673) and a *p* value of 0.00064.

**Conclusions:**

In our study, steroid use delayed the progression of dementia. Further study is needed to outline how steroids and anti‐inflammatory medications can be used in the treatment and prevention of AD.

## Graphical abstract

Figure shows the Kaplan–Meier curves for the group that used systemic steroids and the group that did not use systemic steroids. The time is in days, and the number of risk tables is presented with the curves. The *P* value for the hazard ratio is 0.001. In our study, steroid use delayed the progression of dementia. The hazard ratio (HR) comparing the group using steroids to those not using steroids was 0.26 with a 95% CI of (0.1013, 0.673) and a *p* value of 0.001.

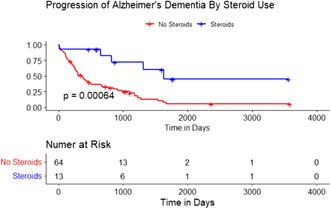

## 1 Introduction

Dementia is a heterogeneous group of syndromes that result in a severe long‐term decline in cognitive function that is significant enough to affect daily function. The most common form of dementia is Alzheimer's disease [1]. An estimated 6.9 million Americans aged 65 and older are living with Alzheimer's dementia today. This number could grow to 13.8 million by 2060, barring the development of medical breakthroughs to prevent or cure this disease [2].

Alzheimer's disease starts with the accumulation of beta‐amyloid protein fragments forming plaques outside of neurons in the brain and tau protein forming neurofibrillary tangles within neurons, leading to inflammation and cortical atrophy [3].

Progression occurs on a spectrum, of which the significant phases are preclinical Alzheimer's disease, mild cognitive impairment (MCI) due to Alzheimer's disease, and dementia due to Alzheimer's disease. The dementia phase itself is further divided into mild, moderate, and severe based on symptomatic presentation. Progression through the stages is variable and influenced by age, genetics (particularly the e4 APOE gene), race, medical comorbidities, physical activity, smoking, and alcohol use. The preclinical phase has no physical symptoms, is diagnosed primarily by the presence of biomarkers, such as beta‐amyloid and tau in cerebrospinal fluid or PET scans, and may last many years before the emergence of clinical symptoms. MCI, due to Alzheimer's disease, involves subtle declines in memory, language, and thought processes, which may not be noticeable to the patient or interfere with the patient's activities of daily living (ADLs) [4, 5].

Dementia due to Alzheimer's disease involves more noticeable declines in memory and language. In mild Alzheimer's dementia, individuals can function independently but may require essential assistance with specific daily tasks. In moderate Alzheimer's dementia, which is the longest stage of dementia progression for most patients, individuals experience increasing difficulty with memory and language, along with confusion. Incontinence, insomnia, personality changes, behavioral disturbances, and agitation are all symptoms associated with moderate Alzheimer's dementia [3]. This stage necessitates increased levels of personal assistance. In the final severe stage of dementia, communication ability is greatly impaired, and patients often require higher levels of care due to mobility limitations and the inability to perform ADLs.

The FDA approved a few drugs for treating Alzheimer's disease, including donepezil, rivastigmine, galantamine, memantine, and memantine combined with donepezil [3]. These available memory enhancers are aimed at slowing cognitive decline and do not affect the overall course of the disease, nor do they affect the underlying pathological changes in the brain. The dosages of these agents may be increased as the patient's disease progresses over time. Additional medications, such as mood stabilizers and antipsychotics, have been used for behavioral and psychiatric concerns associated with AD, which may be indicated during the advancement of the disease.

Due to the neural inflammation from activated microglia involved in Alzheimer's disease [6, 7], we hypothesize that the anti‐inflammatory action of steroids could affect the progression of Alzheimer's dementia in patients who have been treated with steroids compared to patients who have not been treated.

## 2 Methods

A retrospective chart review was carried out at the LSUHSC‐NO Healthcare Network Clinic, Uptown‐St. Charles Ave., and University Medical Center of New Orleans to identify patients with Alzheimer's dementia diagnosed between 2012 and 2020 using the ICD codes for Alzheimer's disease: G30.9, F02.80, G30.0, and F02.80. We reviewed prescribed medications, mainly focusing on memory enhancers (donepezil, memantine, rivastigmine, and namzaric), benzodiazepines (alprazolam, clonazepam, clorazepate, diazepam, and lorazepam), mood stabilizers (divalproex), antipsychotics (risperidone, quetiapine, haloperidol, and ziprasidone), and systemic steroids (prednisone, methylprednisolone, hydrocortisone, and dexamethasone) for the date of prescription, date of any dose changes (increase or decrease), and date of discontinuation. The population was subsequently stratified as those patients who have a history of systemic steroid use of any steroids > 3 months, pulse dose steroids (any steroids > 250 mg at least for a day) within 5 years of Alzheimer's dementia diagnosis, or patients who are not on steroids. Subjects were followed until disease progression, the last documented visit, or death, whichever came first per the electronic medical record review. Progression was defined as escalating doses or the addition of memory enhancers and the addition of benzodiazepines, antipsychotics, or mood stabilizers to treat behavioral disturbances of dementia. Subjects were excluded if they had a diagnosis of other types of dementia such as vascular dementia, pseudodementia, Lewy Body dementia, frontotemporal dementia, normal pressure hydrocephalus, and overlapping types of dementia features, or not enough information was available to review drug prescription information and progression of the disease thoroughly. Power and sample size calculations were not conducted prior to data gathering analysis. We used a Cox proportional hazards model to evaluate the difference in time to progression of Alzheimer's disease between those who used steroids and those who did not. We adjusted the analysis for a propensity score using the year of birth, hemoglobin within a year of dementia diagnosis, presence of diabetes mellitus, hypertension, hyperlipidemia, alcohol use, tobacco use, and clinical findings (incontinence, agitation, behavioral disturbance, and insomnia). Study data were collected and managed using REDCap electronic data capture tools hosted by Louisiana State University Health New Orleans [8, 9]. REDCap (Research Electronic Data Capture) is a secure, web‐based software platform designed to support data capture for research studies, providing (1) an intuitive interface for validated data capture, (2) audit trails for tracking data manipulation and export procedures, (3) automated export procedures for seamless data downloads to standard statistical packages, and (4) procedures for data integration and interoperability with external sources. Study data were analyzed using R (version 4.2.2; R Foundation for Statistical Computing). The Institutional Review Board of Louisiana State University Health Sciences Center in New Orleans, Louisiana deemed the study exempt (Protocol #1441).

## 3 Results

Using the ICD codes mentioned above, 459 patients were identified. Of the 459 patients, 77 met the inclusion criteria. Of the 77 patients included in the study, 64 did not use steroids, and 13 did use steroids. Of the 77 patients included in the study, 59 had progression of their disease, and of those, five used steroids. The median time to progression was 408.00 (191.00, 979.00) days for the overall sample. The median time to progression for the group that did not use steroids was 319.50 (154.00, 817.00) days and 872.00 (597.00, 1632.00) days for the group that did use steroids. The characteristics of the patients overall and divided by steroid use are presented in Table 1. The median duration of steroid use was 25.50 (6.75, 99.75) days. Overall, the participants in the study were born between 1918 and 1977. There were fewer males than females in the overall sample, but males were overrepresented in the group taking steroids (61.5%). Still, this difference was not statistically significant in this small sample (*p* value = 0.195). Black or African Americans were overrepresented in our study, comprising 55.8% of the sample. Most of the study participants had hypertension (92.2%). Of the indicators of dementia stage, incontinence was the most frequent (50.6%) and behavioral disturbances the least reported. Participants using steroids were significantly more likely not to use antipsychotics (*p* value = 0.029). The hazard ratio (HR), adjusted for propensity score, comparing the group using steroids to those not using steroids was 0.26 with a 95% CI of (0.1013, 0.673) and a *p* value of 0.001.

**TABLE 1:** Characteristics of the overall sample and divided based on steroid use.

	Overall	No steroids	Steroids	*p*
n	77	64	13	
Age of birth				0.478
1918–1927	4 (5.2)	4 (6.2)	0 (0.0)	
1928–1937	30 (39.0)	24 (37.5)	6 (46.2)	
1938–1947	29 (37.7)	26 (40.6)	3 (23.1)	
1948–1957	11 (14.3)	8 (12.5)	3 (23.1)	
1958–1967	2 (2.6)	1 (1.6)	1 (7.7)	
1968–1977	1 (1.3)	1 (1.6)	0 (0.0)	
Sex = Male (%)	32 (41.6)	24 (37.5)	8 (61.5)	0.195
Race (%)				0.655
White	27 (35.1)	21 (32.8)	6 (46.2)	
Black or African American	43 (55.8)	37 (57.8)	6 (46.2)	
Other	7 (9.1)	6 (9.4)	1 (7.7)	
Year of Alzheimer's diagnosis = 2016–2021 (%)	47 (61.0)	39 (60.9)	8 (61.5)	1
Hemoglobin (median [IQR])	12.50 [11.30, 13.60]	12.55 [11.80, 13.60]	11.60 [10.20, 13.50]	0.189
Hypertension = yes (%)	71 (92.2)	58 (90.6)	13 (100.0)	0.56
Diabetes mellitus = yes (%)	34 (44.2)	27 (42.2)	7 (53.8)	0.642
Smoking = yes (%)	36 (46.8)	28 (43.8)	8 (61.5)	0.386
Alcohol use = yes (%)	11 (14.3)	8 (12.5)	3 (23.1)	0.576
Insomnia = yes (%)	38 (49.4)	32 (50.0)	6 (46.2)	1
Incontinence = yes (%)	39 (50.6)	33 (51.6)	6 (46.2)	0.959
Behavioral disturbance = yes (%)	26 (33.8)	24 (37.5)	2 (15.4)	0.224
Agitation = yes (%)	36 (46.8)	33 (51.6)	3 (23.1)	0.116
Donepezil = yes (%)	52 (67.5)	42 (65.6)	10 (76.9)	0.64
Memantine = yes (%)	32 (41.6)	28 (43.8)	4 (30.8)	0.577
Rivastigmine = yes (%)	5 (6.5)	4 (6.2)	1 (7.7)	1
Namzaric = yes (%)	4 (5.2)	2 (3.1)	2 (15.4)	0.258
Benzodiazepine = yes (%)	27 (35.1)	20 (31.2)	7 (53.8)	0.216
Antipsychotics = yes (%)	36 (46.8)	34 (53.1)	2 (15.4)	0.029
Mood stabilizers = yes (%)	3 (3.9)	3 (4.7)	0 (0.0)	0.992

*Note:* This table outlines the demographic and clinical characteristics, including medication use for the overall sample, and is divided based on systemic steroid use.

## 4 Discussion

Alzheimer's disease is a multifactorial age‐related [10] neurodegenerative disease with limited treatment options to prevent its progression. This has prompted multiple studies on the risk factors for Alzheimer's disease in the hopes of identifying those at risk early to target prevention and early detection. Neuroinflammation was historically considered a secondary event in neurodegenerative diseases. Accumulating evidence now suggests that it is a critical mechanism in Alzheimer's disease initiation and progression. Abnormalities of Aβ and tau cause activation of microglia and astrocytes and trigger the innate immune system by releasing proinflammatory mediators [10, 11, 12]. Therefore, glial activation with associated inflammatory mediators and regulators emerged as important targets for therapeutic approaches in Alzheimer's disease research [13]. Preclinical evidence indicates that neuro and sex steroids can promote neuronal survival, neurogenesis, and memory function by limiting apoptosis, oxidative stress, mitochondrial failure, and microglial activation. These findings have prompted interest in research evaluating the use of nonsteroidal anti‐inflammatory drugs (NSAIDs) and commonly prescribed corticosteroids such as prednisone, hydrocortisone, methylprednisolone, and dexamethasone in the treatment and prevention of Alzheimer's disease [7, 14, 15], but results have been inconsistent. For example, an autopsy study by Beeri et al. [16], a co‐twin control study by Breitner et al. [17], and a perspective piece by Alisky [18] suggested the benefits of steroid use. However, a placebo‐controlled randomized trial of low‐dose prednisone in Alzheimer's disease did not find any evidence of benefit. In that clinical trial, the active treatment regimen consisted of an initial dose of 20 mg of prednisone daily for 4 weeks tapered to a maintenance dose of 10 mg daily for 1 year, followed by gradual withdrawal during an additional 16 weeks and the primary outcome measure was 1‐year change in the cognitive subscale of the AD Assessment Scale [19]. In our study, the use of multiple kinds of oral steroids was included, but median duration of steroid use was only 25.50 days, much shorter than the duration used in the clinical trial raising concerns for possible unaccounted for confounding that explains the difference between groups despite the use of propensity scoring. Moreover, we did not adjust for the use of NSAIDs, which is challenging to gather from retrospective chart review as many NSAIDs are bought over the counter and not always captured in electronic medical record documentation. A study of elderly humans demonstrated that elevated cortisol levels were associated with memory defects and atrophy of the hippocampus, a structure critical for memory [20]. Our study was limited by the retrospective nature of the analysis from electronic medical records, which are not collected for primary research purposes, and the small sample size. Larger sample sizes could be obtained from high‐quality cohorts; this is a potential next step for the authors in studying this topic. The use of propensity scoring strengthens our study as we were able to adjust for more variables than our sample size would typically allow, and the overrepresentation of African American patients in our study provides important new data regarding the progression of Alzheimer's disease in this often underrepresented and underserved population.

## Author Contributions

All authors were responsible for reviewing and drafting the manuscript. S.B., E.A., and L.A. were responsible for protocol design. A.C., L.C., A.M., and J.Z.C. were responsible for data collection. J.Z.C. was responsible for statistical analysis.

## Ethics Statement

The study was approved using an exempt procedure by the Louisiana State University Health Sciences Center Institutional Review Board (Protocol # 1441).

## Consent

The authors have nothing to report.

## Conflicts of Interest

The authors declare no conflicts of interest.
